# Regional and racial/ethnic inequalities in public drinking water fluoride concentrations across the US

**DOI:** 10.1038/s41370-023-00570-w

**Published:** 2023-06-30

**Authors:** Rose Hefferon, Dana E. Goin, Jeremy A. Sarnat, Anne E. Nigra

**Affiliations:** 1 Department of Environmental Health, Emory University Rollins School of Public Health, Atlanta, GA USA; 2 Program on Reproductive Health and the Environment, Department of Obstetrics, Gynecology, and Reproductive Sciences, University of California, San Francisco, CA USA; 3 Department of Environmental Health Sciences, Columbia University Mailman School of Public Health, New York, NY USA

**Keywords:** Fluoride, Drinking water, Environmental justice

## Abstract

**Background:**

Although the US Centers for Disease Control and Prevention (CDC) considers fluoridation of community water systems (CWSs) to be a major public health achievement responsible for reducing dental disease, recent epidemiologic evidence suggests that chronic exposure to population-relevant levels of fluoride may also be associated with adverse child neurodevelopmental outcomes. To our knowledge, a nationally representative database of CWS fluoride concentration estimates that can be readily linked to US epidemiologic cohorts for further study is not publicly available. Our objectives were to evaluate broad regional and sociodemographic inequalities in CWS fluoride concentrations across the US, and to determine if county-level racial/ethnic composition was associated with county-level CWS fluoride.

**Methods:**

We generated CWS-level (*N* = 32,495) and population weighted county-level (*N* = 2152) fluoride concentration estimates using over 250,000 routine compliance monitoring records collected from the US Environmental Protection Agency’s (EPA) Third Six Year Review (2006–2011). We compared CWS-level fluoride distributions across subgroups including region, population size served, and county sociodemographic characteristics. In county-level spatial error models, we also evaluated geometric mean ratios (GMRs) of CWS fluoride per 10% higher proportion of residents belonging to a given racial/ethnic subgroup.

**Results:**

4.5% of CWSs (serving >2.9 million residents) reported mean 2006–2011 fluoride concentrations ≥1500 µg/L (the World Health Organization’s guideline for drinking water quality). Arithmetic mean, 90^th^, and 95^th^ percentile contaminant concentrations were greatest in CWSs reliant on groundwater, located in the Southwest and Eastern Midwest, and serving *Semi-Urban, Hispanic* communities. In fully adjusted spatial error models, the GMR (95% CI) of CWS fluoride per a 10% higher proportion of county residents that were Hispanic/Latino was 1.16 (1.10, 1.23).

**Impact statement:**

We find that over 2.9 million US residents are served by public water systems with average fluoride concentrations exceeding the World Health Organization’s guidance limit. We also find significant inequalities in community water system fluoride concentration estimates (2006–2011) across the US, especially for Hispanic/Latino communities who also experience elevated arsenic and uranium in regulated public drinking water systems. Our fluoride estimates can be leveraged in future epidemiologic studies to assess the potential association between chronic fluoride exposure and related adverse outcomes.

## Introduction

Fluoridation of public water systems is effective at reducing dental caries and is considered a major US public health achievement [1, 2]. The overall prevalence of dental caries has significantly declined since the initiation of water system fluoridation in 1945, even in communities without fluoridated water [2]. Yet, dental caries remain one of the most prevalent adverse health outcomes for US adolescents and affect one-quarter of children living below the federal poverty level [3]. Significant disparities in the prevalence, severity, and treatment of dental caries persist across racial/ethnic and socioeconomic groups [4–6]. To address these disparities, the US Centers for Disease Control and Prevention’s (CDC) Healthy People 2030 goals include increasing the population served by optimally fluoridated water (defined as 700 µg/L) from 72.8% (current estimate) to 77.1% [7].

However, chronic exposure to high levels of fluoride (>4000 µg/L), especially in childhood, is also associated with skeletal fluorosis and severe enamel fluorosis [8]. Emerging evidence suggests that chronic exposure to water fluoride ranging from <300–1200 µg/L (including concentrations considered optimal by CDC) is also associated with adverse neurodevelopmental outcomes such as reduced intelligence quotient (IQ) scores in children [9–12]. These associations may differ based on timing of exposure across prenatal, infancy, and childhood periods [13]. Several studies have also evaluated the association between chronic exposure to population-relevant levels of fluoride and adverse birth outcomes, endocrine system disruption, and increased risk of bone fractures, although findings for these outcomes are more inconsistent and epidemiologic studies are limited [12–18]. Although achieving optimal levels of fluoride in drinking water is considered critical by CDC to both preventing dental decay and avoiding adverse outcomes, further high-quality epidemiologic studies are needed at relevant concentrations to characterize these associations in US populations [8, 19].

Public water system fluoridation is widespread and is the major source of fluoride exposure in the US, accounting for an estimated 40–70% of exposure in children and 60% in adults [19]. The majority of US residents rely on public drinking water systems, with over 90% receiving some water from community water systems (CWSs) which serve the same populations year-round and are regulated by the US Environmental Protection Agency (EPA) [20]. In response to emerging evidence that chronic exposure to high concentrations of fluoride is associated with skeletal and dental fluorosis and other adverse outcomes, the current US EPA maximum contaminant level (MCL) for fluoride has been gradually reduced over several decades from its original standard of 12,000 µg/L (1962) to the current standard of 4000 µg/L [8]. However, the World Health Organization’s (WHO) Guidelines for Drinking-Water Quality (GDWQ) level for fluoride is much lower at 1500 µg/L. Given that some water sources contain high levels of naturally occurring geogenic fluoride, WHO also recommends that some localities consider reducing drinking water fluoride concentrations even further to <1500 µg/L [21]. Moreover, the US Public Health Service (USPHS) recommends an optimal fluoride concentration of 700 µg/L for manually fluoridated systems to prevent tooth decay while minimizing the risk of dental fluorosis [19, 22].

The emerging evidence supporting an association between chronic, population-relevant levels of fluoride exposure and adverse child neurocognitive health outcomes raises concerns for US communities with high (>1200 µg/L) concentrations of fluoride in drinking water [23]. Previous studies indicate that CWSs serving communities that are majority Hispanic/Latino or American Indian, small and located in rural areas, and those located in the Southwest and Central Midwest have higher concentrations of other inorganic chemicals in drinking water including arsenic, uranium, and nitrates [24–26]. At the county-level, higher proportions of Hispanic/Latino residents are associated with higher county-level CWS arsenic and uranium concentrations [27]. Inequalities in CWS fluoride concentrations have not been similarly evaluated across the US. To our knowledge there are currently no publicly available, nationwide estimates of fluoride concentrations in public drinking water systems across the US that can be easily leveraged for such epidemiologic studies.

The objectives of this study were to (a) characterize broad sociodemographic inequalities in CWS-level fluoride concentrations, and (b) to determine if county-level racial/ethnic composition was associated with county-level CWS fluoride concentrations. At the CWS level (objective 1), we evaluated the following subgroups in our analysis: US region, sociodemographic county clusters, population-served size, source water type, and CWSs which serve correctional facilities. Given prior findings for other inorganic contaminants (uranium and arsenic), we anticipated that a higher county-level proportion of Hispanic/Latino residents would also be associated with higher county-level CWS fluoride concentrations (objective 2).

## Materials And methods

### CWS-level fluoride concentration estimates

To develop CWS-level fluoride concentration estimates, we used 2006–2011 routine compliance monitoring records published in the US EPA’s database supporting the Third Six Year Review (SYR3 database), following a protocol previously developed by our team and published for CWS-level estimates of other regulated inorganic contaminants including arsenic and uranium [25, 26, 28]. The SYR3 database contains records voluntarily submitted by states and other primacy agencies to EPA and is the largest compliance monitoring dataset ever compiled by US EPA. Records represent over 95% of public water systems which serve a total of 290 million people annually (92% of the total population served by public water systems nationwide) [29]. Data from 46 states, Washington, D.C., and American Indian tribes (including those in EPA Regions 1, 4, 5, 8, 9, and Navajo Nation) were submitted for inclusion in the SYR3 (Colorado, Delaware, Georgia, Mississippi, and tribal systems from EPA Regions 2, 6, 7, and 10 did not submit data). All data management and analysis was conducted in R (v 4.1.1) [30].

From a total of *N* = 256,237 fluoride monitoring records, we developed fluoride concentration estimates for *N* = 32,495 CWSs. A total of 178,704 (69.7%) records reported values above the limit of detection (LOD). Fluoride concentrations below the LOD were replaced by the record-specific LOD divided by the square root of two, as previously described [25]. Monitoring records included both treated (i.e. finished) and raw (i.e. untreated) samples, and some CWS report both. We first averaged CWS-level concentrations within the calendar year, and calculated the yearly averages with only treated water samples when yearly averages for untreated samples were higher (to reflect fluoride concentrations distributed to consumers). Fluoride concentrations were reported in both µg/L and mg/L, and we converted concentrations to µg/L to enable direct comparisons with previously published estimates of average CWS and county-level concentrations of other regulated inorganic contaminants (i.e. arsenic, uranium, and others) [25, 26]. We then averaged CWS fluoride concentrations to the overall 2006–2011 time period to estimate chronic (six-year) concentration estimates. EPA’s compliance monitoring framework requires CWSs to collect compliance monitoring records at least once every three years (yearly for surface water systems and once every three years for groundwater systems) [28, 31]; averaging to alternative time periods that correspond directly with the compliance monitoring framework yielded similar findings (Supplemental Fig. 1, Supplemental Table 1). Six-year average CWS fluoride concentrations were then merged with other descriptive information for each CWS from EPA’s Safe Drinking Water Information System (SDWIS) database, including the county/counties-served, the size of the population served (standard EPA categories, ≤500 persons, 501–3300 persons, 3301–10,000 persons, >10,000 persons), whether the system was managed by a tribal authority, source water type (any groundwater versus surface water only), and whether the CWS exclusively served correctional facilities (identified via a keyword search as defined in Table 1) [32]. Based on the county-served by the CWS, we assigned CWSs to (a) US regions (previously categorized as Alaska/Hawaii, Central Midwest, Eastern Midwest, Mid-Atlantic, New England, Pacific Northwest, Southeast, Southwest, defined in Table 1) and (b) to broad sociodemographic county-clusters that were previously developed by a different research group to enable the comparison of county-level outcomes while accounting for sociodemographic makeup of county population (categorized as *Semi-Urban, High Socioeconomic Status (SES); Semi-Urban, Mid/Low SES; Semi-Urban, Hispanic; Mostly Rural, Mid-SES; Rural, Mid/Low SES; Young, Urban, Mid/High SES; Rural, American Indian; and Rural, High SES*) [26, 33]. To determine if a CWS reported manual fluoridation, we extracted manual fluoridation information for each CWS from the CDC’s My Water’s Fluoride database for *N* = 42 states (*N* = 25,792 CWSs in our database) which voluntarily contributed information. Data was not available for tribal CWSs and those from HI, MT, NJ, NM, OH, SD, WA, or WY [34].

**Table 1:** Mean, 90^th^ percentile, and 95^th^ percentile of fluoride concentrations (µg/L) in community water systems (CWSs) and the number and percentage of CWSs exceeding regulatory and guidance levels, nationwide and stratified by subgroup from 2006–2011 (*N* = 32,495 CWSs).

	*N*	*N* (%)> US EPA MCL 4000 µg/L	*N* (%)> WHO GDWQ 1500 µg/L	*N* (%)> USPHS 700 µg/L	Mean, µg/L (95% CI)	90% (95%) percentiles, µg/L
All CWSs	32,495	99 (0.3%)	1456 (4.5%)	4,992 (15.4%)	376 (370, 383)	1000 (1428)
Total population	184,802,919	40,163	2980,013	20,539,615		
Source water type						
Groundwater^a^	29,928	99 (0.3%)	1443 (4.8%)	4815 (16.1%)	388 (381, 395)	1040 (1485)
Surface water	2567	0 (0%)	13 (0.5%)	177 (6.9%)	240 (227, 253)	651 (914)
*P*-value						<0.001
Size of population served^b^						
≤500	19,436	81 (0.4%)	920 (4.7%)	2716 (14%)	354 (345, 362)	939 (1460)
500–3300	7827	17 (0.2%)	368 (4.7%)	1392 (17.8%)	410 (396, 423)	1177 (1475)
3301–10,000	2806	1 (0%)	110 (3.9%)	500 (17.8%)	411 (392, 429)	1115 (1380)
10,001–100,000	2136	0 (0%)	55 (2.6%)	359 (16.8%)	411 (392, 431)	971 (1260)
>100,000	290	0 (0%)	3 (1%)	25 (8.6%)	387 (342, 431)	681 (851)
*P*-value						<0.001
Region^c^						
Alaska/Hawaii	418	0 (0%)	1 (0.2%)	7 (1.7%)	85 (66, 103)	250 (409)
Central Midwest	2436	5 (0.2%)	94 (3.9%)	404 (16.6%)	415 (394, 435)	1044 (1406)
Eastern Midwest	4887	3 (0.1%)	260 (5.3%)	1336 (27.3%)	524 (509, 540)	1295 (1530)
Mid-Atlantic	3641	1 (0%)	15 (0.4%)	100 (2.7%)	114 (103, 125)	329 (535)
New England	1634	0 (0%)	77 (4.7%)	252 (15.4%)	323 (295, 350)	1025 (1479)
Pacific Northwest	3848	4 (0.1%)	100 (2.6%)	313 (8.1%)	254 (240, 269)	650 (1000)
Southeast	7107	27 (0.4%)	271 (3.8%)	734 (10.3%)	310 (297, 323)	720 (1271)
Southwest	8524	59 (0.7%)	638 (7.5%)	1846 (21.7%)	527 (512, 542)	1277 (1880)
*P*-value						<0.001
Sociodemographic county cluster^d^						
Semi-Urban, High SES	12,531	8 (0.1%)	349 (2.8%)	1551 (12.4%)	306 (298, 315)	836 (1233)
Semi-Urban, Mid/Low SES	1325	8 (0.6%)	69 (5.2%)	194 (14.6%)	376 (343, 410)	969 (1524)
Semi-Urban, Hispanic	4536	50 (1.1%)	391 (8.6%)	1122 (24.7%)	605 (582, 628)	1376 (2020)
Mostly Rural, Mid-SES	7837	9 (0.1%)	327 (4.2%)	1030 (13.1%)	322 (310, 334)	930 (1400)
Rural, Mid/Low SES	499	0 (0%)	24 (4.8%)	93 (18.6%)	377 (337, 418)	1019 (1403)
Young, Urban, Mid/High SES	1022	2 (0.2%)	12 (1.2%)	67 (6.6%)	254 (231, 277)	550 (829)
Rural, American Indian	437	1 (0.2%)	18 (4.1%)	86 (19.7%)	432 (381, 484)	1042 (1348)
Rural, High SES	4448	21 (0.5%)	269 (6%)	879 (19.8%)	457 (438, 476)	1270 (1649)
*P*-value				1551 (12.4%)		<0.001
Correctional facility CWSs	192	0 (0%)	5 (2.6%)	23 (12%)	294 (234, 354)	753 (1111)
*P*-value						0.058
Manual fluoridation (available for *N* = 25,617 CWSs)^e^	6130	62 (1.0%)	997 (16.3%)	3295 (53.8%)	973 (953, 992)	1886 (2515)

*EPA* Environmental Protection Agency, *MCL* maximum contaminant level (4000 µg/L). *WHO GDWQ* World Health Organization guideline for drinking water quality (1500 µg/L). *USPHS* United States public health service, recommended optimal water fluoride concentration (700 µg/L).

*P*-values are from non-parametric Kruskal–Wallis test.

^a^CWSs served by groundwater include those served by surface water under the influence of groundwater and groundwater under the influence of surface water.

^b^Categories of population served are standard U.S. EPA categories. Population served is adjusted total population served, which accounts for systems that sell or purchase water and avoids overcounting.

^c^States included in geologic regions are: Alaska/Hawaii (AK, HI), Central Midwest (ND, SD, NE, KS, MO), Eastern Midwest (WI, IL, IN, MI, OH, MN, IA), Mid-Atlantic (PA, MD, DC, DE, NY, NJ, CT, RI), New England (MA, VT, NH, ME), Pacific Northwest (WA, OR, MT, WY, and ID), Southeast (OK, AR, LA, MS, AL, FL, GA, TN, KY, SC, NC, VA, WV), and Southwest (CA, NV, UT, CO, AZ, NM, TX).

^d^Very few CWSs served more than one county; of these, approximately half served counties categorized to different sociodemographic county-clusters (e.g., NY7003493) serves New York, New York (Young, Urban, Mid/High SES) and Bronx, New York (Semi-Urban, Hispanic). Sociodemographic clusters were classified based on Wallace et al. [33]. These CWSs are represented for each county that they serve in the sociodemographic county-cluster analyses (*N* = 32,653).

^e^Voluntarily reported by states to CDC’s My Water’s Fluoridation database.

### Population-weighted, county-level CWS fluoride concentrations (independent variable)

We next aggregated CWS fluoride estimates to the county-level, as previously described in detail [25, 26]. We created mean, 90^th^, and 95^th^ percentile county CWS fluoride concentrations, weighted by the size of the population served by each CWS serving a given county. The population-served weight for each CWS was calculated as the population served by that CWS divided by the total population served by all CWSs serving that county. We treated county estimates as missing when CWSs serving that county cumulatively reported serving less than fifty percent of the public water-reliant population in the entire county (*N* = 374 out of 2526 counties with available data, for a final sample size of *N* = 2152 counties) [35, 36]. We also calculated the percent of the population served by CWSs within a county that was served by CWSs which reported manual fluoridation. We mapped county-level CWS fluoride concentrations using the *maps* R package [37]. We were unable to aggregate CWS-level data to a finer geographic resolution (e.g. zip code) because only county-served was consistently and reliably reported in SDWIS for all states, as previously described [25].

### County-level sociodemographic variables (dependent variables)

To determine if county-level racial/ethnic composition was associated with county-level CWS fluoride concentrations, we merged county-level CWS fluoride concentrations with several county-level sociodemographic variables. We derived the following county-level variables from the 2010 decennial US Census: total population, population density (population per square mile), and racial/ethnic composition, including the total number and proportion of residents who identified as American Indian or Alaskan Native (hereafter referred to as American Indian/Alaskan Native), non-Hispanic Asian, Native Hawaiian or Other Pacific Islander, Hispanic/Latino of any race, non-Hispanic Black or African American (hereafter referred to as non-Hispanic Black), and non-Hispanic White [38]. To assess county-level socioeconomic vulnerability, we downloaded the 2014 Centers for Disease Control (CDC)/Agency for Toxic Substances and Disease Registry (ATSDR)’s county-level social vulnerability index for socioeconomic status [39]. This index is derived from 2010–2014 American Community Survey estimates of median household income and the percent of adults who are unemployed, living below the poverty line, and without a high school diploma (higher scores indicate higher socioeconomic vulnerability). We also downloaded the percent of adults with a high school diploma and median household income for sensitivity analyses (derived from the US Census American Community Survey and the Small Area Income and Poverty Estimates/National Center for Education Statistics data, respectively) [40, 41]. We estimated the percent of public drinking water supplied by groundwater sources (versus surface water) from estimates of total groundwater and surface water withdrawn for public drinking water calculated by the US Geological Survey for 2010, as previously described [42, 43].

### Statistical analysis: inequalities in CWS-level fluoride concentrations

To evaluate inequalities in fluoride concentrations at the CWS-level (our first objective), we compared arithmetic mean (95% confidence interval), 90^th^, and 95^th^ percentile fluoride concentrations and the number and percent of CWSs with concentrations exceeding the EPA MCL (4000 µg/L), the WHO GDWQ (1500 µg/L), and the current USPH recommended level (700 µg/L, previously 700–1000 µg/L) across source water type, size of population served, correctional facilities, US region, and broad sociodemographic county-clusters. We evaluated both the mean and 90^th^ (95^th^) percentile concentrations because (a) measures of central tendency are influenced by the high proportion of records at or below the LOD, while higher percentile values are not, and (b) measures of central tendency do not reflect percentiles at the highest ends of the distribution which reflect the most highly exposed populations and are most influenced by regulatory action to reduce exposures [26, 44]. We assessed statistical significance for differences in fluoride concentration distributions across subgroups via non-parametric Kruskal–Wallis tests.

### Statistical analysis: county-level racial/ethnic composition and CWS fluoride

We next evaluated the association between county-level racial/ethnic composition and CWS fluoride concentrations (our second objective). From a total of 2126 conterminous US counties with CWS fluoride estimates available (out of 3108 conterminous counties/county equivalents in the 2010 US Census), we excluded 17 counties missing the percent of public water sourced from groundwater, and 1 county missing socioeconomic vulnerability index score for a sample size of *N* = 2108 counties. No counties were missing Census racial/ethnic composition variables. We further restricted our analyses to counties with at least 100 residents in each racial/ethnic group of interest to avoid violating the positivity assumption and extrapolating beyond the range of observed data, and to minimize bias that could result if small changes in the absolute number of residents yielded large relative percentage differences. As a result, sample sizes for the analysis for each racial/ethnic group differed. We evaluated differences in CWS fluoride concentrations and sociodemographic characteristics across all counties included in our analysis and those included in the analysis for each racial/ethnic group. We also compared characteristics across counties excluded from our analyses to assess potential selection bias (Supplemental Table 2).

We assessed spatial autocorrelation (dependence) in county-level CWS fluoride concentrations using Moran’s I (a correlation coefficient assessing global spatial autocorrelation). We defined neighbors using a simple queen contiguity matrix (*i* = 1 for neighbors, *i* = 0 for non-neighbors). Moran’s I indicated significant global spatial autocorrelation (I = 0.48, *p* < 0.001), indicating that effect estimates from ordinary least squares models could be biased. To identify whether a spatial error or spatial lag model would be most appropriate, we conducted a Lagrange Multiplier diagnostic test for spatial dependence for models assessing a 10% higher proportion of residents in each of the racial/ethnic subgroups via the *lagsarlm* function in the “spatialreg” R package [45]. *P*-values for both spatial lag and spatial error parameters were <0.001, and we proceeded using spatial error models because effect estimates were larger for most models we assessed.

We evaluated the geometric mean ratio (GMR), 95% CIs, and corresponding percent differences of county-level CWS fluoride concentrations per 10% higher proportion of residents who were Hispanic/Latino, non-Hispanic Black, American Indian, and non-Hispanic White. We were unable to assess associations for the proportion of residents who were non-Hispanic Asian or Native Hawaiian or Other Pacific Islander because there were very few counties with >100 residents. Model 1 adjusted for the percent of public drinking water served by a groundwater source, population density, and socioeconomic vulnerability index score. To determine if associations with racial/ethnic composition were explained by CWS manual fluoridation, Model 2 further adjusted for the county-level proportion of public drinking water from CWSs which report fluoridation via CDC’s My Water’s Fluoride database, weighted by the population served by each CWS.

We conducted several sensitivity analyses. First, we repeated our analysis using the 95^th^ percentile public drinking water fluoride concentrations (rather than the mean) because average concentration estimates likely underestimate exposures for the most highly exposed groups, with similar findings (Supplemental Table 3). Second, we repeated our analyses assessing a 60% higher proportion of residents in a given racial/ethnic group, which is a common cut-point in the literature to identify majority communities, with similar interpretations although the magnitudes of the effect estimates were magnified in both directions (Supplemental Table 3). Third, we repeated our main models adjusting for median household income and the percent of adults with a high school diploma (rather than the socioeconomic vulnerability index), also with similar findings (Supplemental Table 3). We also modeled the associations using flexible cubic spline models, with knots at the 50th and 90^th^ percentiles and the reference set to the 10^th^ percentile of the county-level CWS fluoride distribution, to assess potential non-linearity in the association. We observed relatively linear associations between higher proportions of residents in each racial/ethnic subgroup and CWS fluoride concentrations and therefore retained linear models. Finally, we repeated our analyses stratified by region in exploratory analyses to assess potential effect measure modification.

## Results

**Fig. 1:**
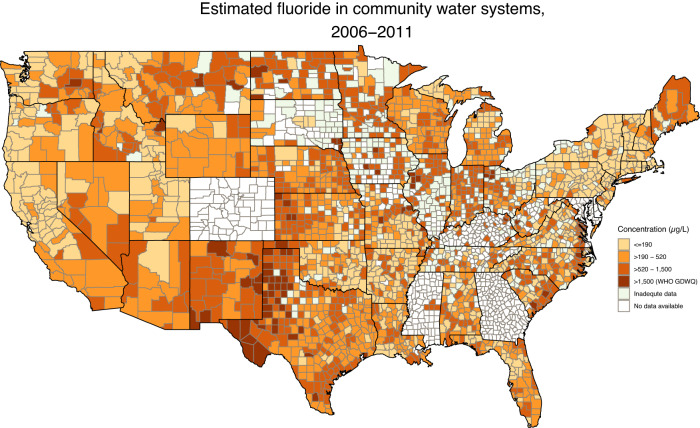
County-level population weighted average of fluoride concentrations in community water systems (CWSs) from 2006–2011 (*N* = 32,495 CWSs serving *N* = 2152 counties). Average concentrations were weighted by the population served by each CWS to estimate the county-level weighted average CWS concentrations. Counties which were not represented by any CWSs in the SYR3 database are labeled as “No data available.” Counties with “Inadequate data” did not have CWS data representing at least 50% of the public water reliant population. The highest concentration category (>1500 µg/L) represents counties with a weighted average fluoride concentration exceeding the World Health Organization’s (WHO) guideline for drinking water quality (two of these counties had weighted averages exceeding the EPA’s maximum contaminant level of 4000 µg/L). The two lowest concentration categories (<= 190 and >190–520 µg/L) divide the remaining counties into two equally sized groups.

We developed 2006–2011 fluoride exposure estimates for 32,495 CWSs (serving a total of 180 million residents, Table 1) and *N* = 2,152 counties (Fig. 1). Of these CWSs, 15.4% (*N* = 4,992 serving a population of >20.5 million) had six-year average fluoride concentrations above the USPHS recommended level (700 μg/L); 4.5% (*N* = 1456, serving a population of >2.9 million) exceeded the WHO GDWQ (1500 μg/L); and 0.3% (*N* = 99, serving a population of >40,000) exceeded the US EPA MCL (4000 μg/L) (Table 1). Nationwide, the mean, 90^th^, and 95^th^ percentiles of estimated CWS fluoride CWSs from 2006–2011 were 376, 1000, and 1428 μg/L (Table 1). Out of 25,617 CWSs voluntarily reporting fluoridation information to CDC, only 6130 (24%) reported manual fluoridation (Table 1), which is much lower than EPA’s estimate that approximately 12,341 CWSs manually fluoridated their water in 2012 [19]. CWSs reporting manual fluoridation through the CDC’s My Water’s Fluoride database were more likely to exceed the WHO GDWQ (16.3%) and the USPHS recommended level (53.8%), but not EPA’s MCL.

CWS fluoride concentrations varied across source water types. CWSs which relied on groundwater had higher mean, 90^th^, and 95^th^ percentile concentrations (388, 1040 and 1485 μg/L) than CWSs which relied on surface water (240, 651, and 914 μg/L) (Table 1; *p* < 0.001). Fluoride concentrations also differed significantly across categories of population served size (*p* < 0.001). The 90th and 95^th^ percentile fluoride concentrations were larger for smaller CWSs serving ≤500 people (939, 1460 μg/L) and medium sized CWSs serving 500–3300 people (1177, 1475 μg/L) and 3301–10,000 people (1115, 1380 μg/L, respectively) than CWSs serving the largest populations with >100,000 people (681, 851 μg/L). Throughout the US, 90^th^, 95^th^ fluoride percentile concentrations (μg/L) for CWSs serving correctional facilities were similar to those for all CWSs (753, 1111 μg/L versus 1000, 1428 μg/L).

CWS fluoride concentrations differed significantly by US region (*p* < 0.001, Table 1). Mean (95% CIs) CWS fluoride concentrations were highest among CWSs in the Southwest (527 μg/L, 95% CI 512, 542), Eastern Midwest (524 μg/L, 95% CI 509, 540) and Central Midwest (415 μg/L, 95% CI 394, 435). Similarly, 90^th^ and 95^th^ percentile fluoride concentrations were highest in the Southwest, Eastern Midwest, and Central Midwest. CWS fluoride concentrations were also significantly different across sociodemographic clusters (*p* < 0.001). Mean (95% CI) fluoride concentrations (μg/L) were highest among CWSs classified as serving *Semi-Urban, Hispanic* counties (605 μg/L, 95% CI 582, 628), followed by CWSs classified as serving *Rural, High SES* counties (457 μg/L, 95% CI 438, 476), and CWSs classified as serving *Rural, American Indian* counties (432 μg/L, 95% CI 381,484) (Table 1). We observed similar rankings when comparing 90^th^ and 95^th^ percentile concentrations.

In county-level analyses assessing the association between county racial/ethnic composition and CWS fluoride concentrations, mean fluoride concentrations were highest for counties included in analyses for non-Hispanic White residents (Table 2). These counties also had a higher percentage of public water sourced from groundwater supplies and the highest percentage of the population living in rural areas. Geometric mean ratios (GMRs) and 95% confidence intervals for county-level CWS fluoride concentrations per 10% higher proportion of residents in each racial/ethnic subgroup are presented in Table 3. In Model 1 adjusted for population density, the percent of public water sourced from groundwater supplies, and socioeconomic vulnerability score, the GMR (95% CI) of CWS fluoride per 10% higher proportion of Hispanic/Latino residents was 1.15 (95% CI 1.09, 1.21), corresponding to a 15% (9%, 21%) change (Table 3). Higher proportions of non-Hispanic White residents were associated with lower CWS fluoride concentrations (GMR 0.84, 95% CI 0.79, 0.90), corresponding to a −16% (−21%, −10%) change. Further adjustment for the percent of public water that was fluoridated (Model 2), produced similar results for higher proportions of both Hispanic/Latino (GMR 1.31, 95% CI 1.21, 1.41) and non-Hispanic White (GMR 0.83, 95% CI 0.78, 0.88) residents. In both models, higher proportions of non-Hispanic Black and American Indian/Alaskan Native residents was not associated with CWS fluoride concentrations (Model 1 GMR 1.01, 95% CI 0.92, 1.12; and GMR 1.07, 95% CI 0.94, 1.21, respectively) (Table 3).

**Table 2:** County-level mean estimated community water system (CWS) fluoride concentrations and sociodemographic characteristics for all counties included in any analysis (*N* = 2108 counties), and separately for counties included in analyses specific to each racial/ethnic group (counties with >100 residents of each racial/ethnic group).

	All counties in any analysis	non-Hispanic Black^a^	American Indian/ Alaskan Native^a^	Hispanic/Latino^a^	non-Hispanic White^a^
*N*	2108	1489	1271	1918	2106
CWS fluoride concentration, µg/L (mean, SD)	503 (506)	464 (465)	436 (420)	496 (493)	503 (506)
Sociodemographic characteristics					
Population size, thousands (mean, SD)	103 (338)	142 (396)	162 (426)	113 (353)	103 (339)
Population density (mean, SD)^b^	158 (550)	217 (645)	236 (690)	173 (575)	158 (550)
% public drinking water sourced from groundwater supplies (mean, SD)^c^	67 (40)	62 (41)	61 (40)	66 (40)	67 (40)
CDC/ATSDR socioeconomic vulnerability index score (mean, SD)	0.48 (0.28)	0.53 (0.27)	0.49 (0.27)	0.5 (0.27)	0.48 (0.28)
Median household income (mean, SD)	44,234 (10,611)	44,900 (11,477)	46,102 (11,517)	44,597 (10,816)	44,238 (10,614)
% adults with high school diploma (mean, SD)	84 (9)	82 (9)	82 (9)	83 (9)	84 (9)
% population living in rural area (mean, SD)	59 (31)	50 (28)	46 (28)	55 (30)	59 (31)
Racial/ethnic composition (mean, SE)					
% non-Hispanic Black	7 (13)	10 (14)	7 (11)	7 (12)	7 (13)
% American Indian/Alaskan Native	2 (7)	1 (4)	3 (8)	2 (6)	2 (7)
% Hispanic/Latino	10 (15)	10 (15)	11 (14)	11 (15)	10 (15)
% non-Hispanic White	79 (19)	75 (19)	76 (18)	78 (19)	79 (19)

*N* = 2108 is the total number of conterminous US counties with data available for CWS fluoride estimates, the percent of public drinking water sourced from groundwater supplies, population density, and CDC/ATSDR socioeconomic vulnerability index score.

^a^N represents the total number of conterminous US counties evaluated for each racial/ethnic group after restricting to counties with >100 residents of the racial/ethnic group of interest.

^b^Population density is calculated as number of residents per square mile.

^c^The percent of public drinking water sourced from groundwater supplies was calculated using nationwide estimates of water use published by the US Geological Survey for 2010.

**Table 3:** Geometric mean ratios (GMR) and 95% CI of county-level community water system (CWS) fluoride concentrations per a 10% higher proportion of residents who are non-Hispanic Black, American Indian/Alaskan Native, Hispanic/Latino, or non-Hispanic White.

	*N*	GMR (95% CI)	Corresponding % change
% non-Hispanic Black	1489		
Model 1		1.01 (0.92, 1.12)	1% (−8%, 12%)
Model 2		0.97 (0.89, 1.07)	−3% (−11%, 7%)
% American Indian/Alaskan Native	1271		
Model 1		1.07 (0.94, 1.21)	7% (−6%, 21%)
Model 2		1.10 (0.94, 1.28)	10% (−6%, 28%)
% Hispanic/Latino	1918		
Model 1		1.25 (1.16, 1.36)	25% (16%, 36%)
Model 2		1.19 (1.10, 1.28)	19% (10%, 28%)
% non-Hispanic White	2106		
Model 1		0.84 (0.79, 0.90)	−16% (−21%, −10%)
Model 2		0.88 (0.82, 0.93)	−12% (−18%, −7%)

Spatial autocorrelation was modeled in spatial error models with autoregressive correlation structure. Model 1 adjusts for population density, the percent of public water sourced from groundwater supplies, and socioeconomic vulnerability score index. Model 2 further adjusts for the percent of public water that was fluoridated as reported in CDC’s My Water’s Fluoridation database.

In exploratory analyses stratified by region, associations were positive but not significant in all regions for higher proportions of Hispanic/Latino residents, and inverse but not significant in all regions for non-Hispanic White participants (Supplemental Table 4). Higher proportions of non-Hispanic Black residents were positively but not significantly associated with higher fluoride concentrations in the Central Midwest, Mid-Atlantic, Southeast, and Southwest, while higher proportions of American Indian/Alaskan Native residents were positively but not significantly associated with higher fluoride concentrations in the Eastern Midwest, Central Midwest, Mid-Atlantic, Pacific Northwest, and Southeast. Stratifying by region resulted in several analyses with a sample size less than 100 counties; these findings should therefore be considered unstable and exploratory.

## Discussion

We found significant inequalities in CWS fluoride concentrations by county sociodemographic characteristics, including by racial/ethnic composition, further raising environmental justice concerns for these communities. Compared to other CWSs, those serving *Semi-Urban, Hispanic* communities and communities in the Southwest were most likely to exceed 700 µg/L (current USPHS optimal concentration), 1500 µg/L (WHO guidance level), and 4000 µg/L (US EPA’s MCL). Our finding that higher proportions of Hispanic/Latino residents was associated with higher average county-level CWS fluoride concentrations adds to a growing body of evidence that Hispanic/Latino communities are disproportionately exposed to higher concentrations of regulated inorganic contaminants in public drinking water, including arsenic, uranium, nitrates, chromium, and selenium [24–27]. For fluoride, this county-level association remained significant even after adjustment for the percent of public water that was manually fluoridated, indicating that naturally occurring fluoride in groundwater sources may be driving higher concentrations for CWSs serving largely Hispanic/Latino communities. In general, inequities in the natural (e.g. hydrogeology, climate), built (e.g. water infrastructure, groundwater reliance), and sociopolitical (e.g. structural racism, social and political vulnerability) environments underlie disparities in public drinking water exposures across the US [46]. Although we identified the highest mean and 90^th^ percentile CWS fluoride concentrations and highest percentage of MCL exceedances in CWSs serving *Semi-Urban, Hispanic* communities, our county-level analysis of racial/ethnic composition did not specifically evaluate if higher proportions of Hispanic/Latino residents was associated with higher fluoride CWS concentrations beyond the USPHS optimal concentration of 700 µg/L. Although this analysis yielded consistent findings across analyses at the CWS and county-level, further analyses are needed at spatial resolutions that more closely reflect community sociodemographic characteristics (e.g. zip code, Census tract).

Substantial spatial variability exists in CWS fluoride concentrations across the US, mirroring spatial patterns in untreated well fluoride concentrations measured by the United States Geological Survey [47]. Similar to previous findings for other inorganic contaminants such as arsenic, chromium, selenium, and uranium, CWSs dependent on groundwater sources and CWSs located in the Southwest had higher estimated fluoride concentration estimates. Fluoride is naturally occurring in US groundwater and concentrations are influenced by well depth, pH, total dissolved solids, Ca/Na molar ratio, alkalinity, water temperature, and precipitation [47]. Hydrogeologic and other environmental factors influencing high concentrations of fluoride in groundwater differ across local contexts. For example, very high (>10,000 µg/L) fluoride concentrations are likely influenced by evaporative concentrations in the Basin and Range basin-fill aquifers in CA, but by geothermal water mixing in the Rio Grande aquifer system in NM [47].

Although CWSs in the US began manually fluoridating public water supplies as early as 1945, to our knowledge our study provides the first nationwide concentration estimates of CWS fluoride across sociodemographic subgroups that can be linked to large, nationwide cohorts across the US for epidemiologic study. CWS fluoridation is widespread; the US Centers for Disease Control and Prevention estimates that 200 million people in the US were served by 12,341 community water systems that manually added fluoride in 2012 [19]. In our analysis, we estimated that over 20 million US residents were served by CWSs with six-year (2006–2011) average fluoride concentrations exceeding the USPHS recommendation for optimal fluoride concentrations in drinking water (700 µg/L). Additional large epidemiologic studies are needed to evaluate if CWS fluoride at and below 700 µg/L is associated with adverse health outcomes in US populations.

Despite several large historical reductions in the EPA’s fluoride MCL and emerging epidemiologic evidence supporting an association between chronic, population-relevant levels of fluoride exposure and child neurocognitive outcomes, epidemiologic studies assessing the association specifically between water fluoride exposure and related health outcomes in US populations remain relatively sparse. Epidemiologic studies of inorganic contaminants often rely on concentrations measured in biospecimens, which often integrate exposures from multiple sources and reflect internal dose. While urine concentrations are considered valid biomarkers of total internal dose for many inorganic contaminants (including fluoride), analyses can be complicated when exposures influence kidney function and urinary excretion [18, 48, 49]. The CWS fluoride concentration estimates derived here can further support epidemiologic studies of fluoride exposure by avoiding these potential biases and reverse causality concerns. Future studies can also evaluate if CWS fluoride concentrations have changed over time in the US, especially in relation to policies promoting fluoridation of public water systems.

Although our study did identify inequalities in CWS fluoride concentrations across sociodemographic groups, our study did not evaluate the association between CWS fluoride concentrations and adverse health outcomes, or inequities in those outcomes. A 2015 Cochrane review concluded that there was insufficient evidence to determine whether fluoridation could reduce current racial/ethnic and socioeconomic inequities in the prevalence, severity, and treatment of dental caries [2]. However, one nationally representative study published in 2019 reported that county-level fluoridation was associated with attenuated income-related inequalities in decayed and filled primary tooth surfaces among children [50].

Our analysis has several limitations. Our evaluation of nationwide CWS fluoride concentrations was limited to CWSs which reported routine compliance monitoring records to EPA’s SYR3 (covering >95% of all public water systems nationwide). Missing compliance monitoring records from states and tribal regions that did not submit compliance monitoring records to the SYR3 (Colorado, Delaware, Georgia, Mississippi, EPA regions 2, 6, 7, and 10) may have biased our findings, especially in analyses stratified by region. In addition, EPA acknowledges inaccuracies and the underreporting of some data reported in SDWIS, and is actively working with states and primacy agencies to improve the quality of the data [51]. Our analysis was also likely limited by inaccuracies in the reporting of manual fluoridation to CDC’s My Water’s Fluoride database [34]. Although EPA estimated that 12,341 CWSs manually fluoridated water in 2012, we only identified 6,130 CWSs in EPA’s SYR3 database with fluoride monitoring records that reported manual fluoridation through the CDC’s My Water’s Fluoride database. Our county-level analyses adjusting for the percentage of public water that was fluoridated may therefore be subject to residual confounding. Moreover, future studies should also assess the association between community water system fluoride concentrations and proximity to military bases, which we were unable to assess in this analysis. Future studies can also leverage the release of EPA’s Six Year Review 4 (not yet released, but covering years 2012–2019) to evaluate if changes in the USPHS’s optimal level of fluoride (lowered from 700–1000 µg/L to 700 µg/L in 2015) was associated with a reduction in CWS fluoride concentrations, and whether these potential reductions were equitable across the US.

One significant limitation of research evaluating inequalities in US public drinking water contaminant concentrations is the lack of a comprehensive, nationwide map of public water system distribution boundaries [52]. Although we have derived both population- and area-weighted average CWS fluoride concentrations at finer resolutions (including zip code, Census tract, block group, and block) for all states which make these distribution boundaries publicly available (e.g. California, Utah), we limited the current analysis to the CWS and county-level because the accuracy of these boundaries have not been fully characterized and boundaries are missing for more than half of all US states. Moreover, distribution boundaries are not publicly available for states within large regions of the US (including the Northern Plains, Midwest, Southeast, and far Northeast areas). Further epidemiologic studies leveraging these fluoride concentration estimates for exposure assignment can assign these estimates at the water system level, zip code level, or county-level, as appropriate for the epidemiologic study of interest.

The current study adds to a growing body of evidence finding higher inorganic contaminant concentrations in CWSs that rely on groundwater, are located in the Southwest, and serve communities with high proportions of Hispanic/Latino residents. Further studies at higher spatial resolution within the Southwest are needed to evaluate whether racial/ethnic inequities in fluoride concentrations differ within this region. Regardless, additional technical, financial, and regulatory support is needed to reduce inorganic contaminant concentrations in CWSs serving these communities, especially for other inorganic contaminants such as arsenic and uranium which have no beneficial role in protecting human health.

### Supplementary information

Datasets

Supplementary Information

## Data Availability

Community water system- and county-level estimates of fluoride concentrations generated as described in this manuscript are included as Supplemental Material.
