# Supplementary material for: Regional and racial/ethnic inequalities in public drinking water fluoride concentrations across the US
Source: J Expo Sci Environ Epidemiol. 2023 Jun 30;34(1):68–76. doi: 10.1038/s41370-023-00570-w (PMC10756931; doi:10.1038/s41370-023-00570-w)
Supplement: Supplementary file 2 — Supplementary Information [file 41370_2023_570_MOESM2_ESM.pdf]

Supplementary Information

Regional and racial/ethnic inequalities in Fig 1. fluoride 2006-2011 white bcg public drinking water fluoride concentrations across the US

Rose Hefferon,[1] Dana E. Goin,[2] Jeremy Sarnat,[1] Anne E. Nigra[3]

[1] Department of Environmental Health, Emory University Rollins School of Public Health
[2] Program on Reproductive Health and the Environment, Department of Obstetrics, Gynecology, and Reproductive Sciences, University of California, San Francisco
[3] Department of Environmental Health Sciences, Columbia University Mailman School of Public Health

Corresponding author: Anne Nigra, ScM PhD; Email: aen2136@cumc.columbia.edu; Address: 722 W 168th St, 11th Floor Rm 1107A, New York, NY 10032

Supplemental Figure 1. County-level population weighted average of fluoride concentrations in community water systems (CWSs) from 2008-2010 (N= 32,495 CWSs serving N= 1,846 counties). Average concentrations were weighted by the population served by each CWS to estimate the county-level weighted average CWS concentrations. Counties which were not represented by any CWSs in the SYR3 database are labeled as “No data available.” Counties with “Inadequate data” did not have CWS data representing at least 50% of the public water reliant population. The highest concentration category (>1,500 µg/L) represents counties with a weighted average fluoride concentration exceeding the World Health Organization’s (WHO) guideline for drinking water quality (two of these counties had weighted averages exceeding the EPA’s maximum contaminant level of 4,000 µg/L). The two lowest concentration categories (<= 190 and >190-520 µg/L) correspond to quantiles splitting the other counties into two equal groups, based on county-level averages from 2006-2011 (see Figure 1 in the main manuscript text).

County–level, population weighted fluoride concentrations in community water systems, 2008–2010

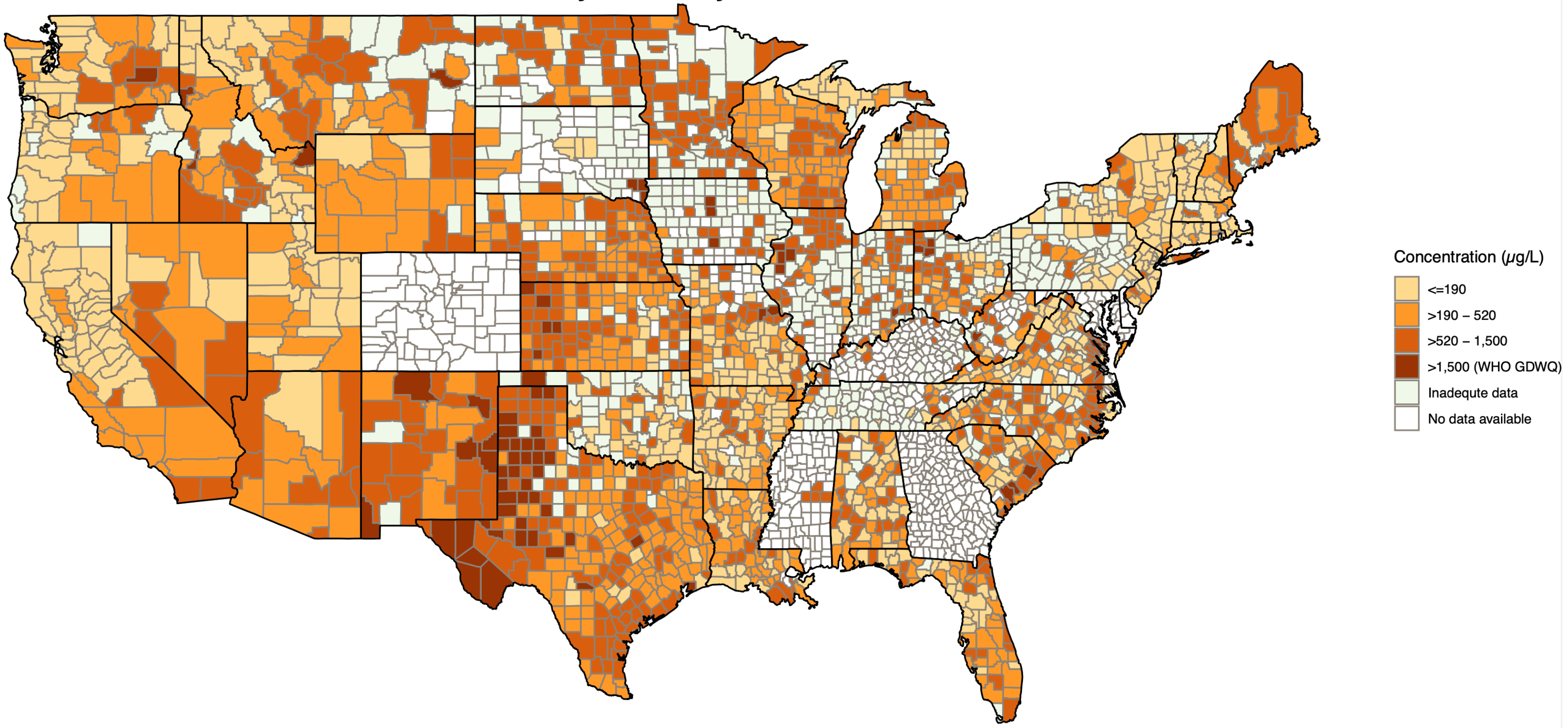

Supplemental Table 1. Average community water system (CWS)-level fluoride concentrations averaged to three (2008-2010) and six (2006-2011) year periods.

	N	Six-year mean (µg/L, 95% CI) 2006-2011	Three-year mean (µg/L, 95% CI) 2008-2010
All CWSs	32,495	376 (370,383)	385 (378,392)
Source water type			
Groundwater [a]	29,928	388 (381,395)	396 (388,403)
Surface water	2,567	240 (227,253)	255 (240,270)
P-value		<0.001	<0.001
Size of population served [b]			
≤500	19,436	354 (345,362)	366 (356,376)
500-3,300	7,827	410 (396,423)	416 (402,430)
3,301-10,000	2,806	411 (392,429)	412 (392,433)
10,001-100,000	2,136	411 (392,431)	403 (382,424)
>100,000	290	387 (342,431)	383 (332,433)
P-value		<0.001	<0.001
Region			
Alaska/Hawaii	418	85 (66,103)	96 (72,119)
Central Midwest	2,436	415 (394,435)	390 (368,412)
Eastern Midwest	4,887	524 (509,540)	536 (519,554)
Mid-Atlantic	3,641	114 (103,125)	112 (103,121)
New England	1,634	323 (295,350)	346 (315,378)
Pacific Northwest	3,848	254 (240,269)	262 (245,279)
Southeast	7,107	310 (297,323)	313 (298,327)
Southwest	8,524	527 (512,542)	535 (518,551)
P-value		<0.001	<0.001
Sociodemographic county cluster [c]			
Semi-Urban, High SES	12,531	306 (298,315)	316 (308,325)
Semi-Urban, Mid/Low SES	1,325	376 (343,410)	351 (318,384)
Semi-Urban, Hispanic	4,536	605 (582,628)	606 (581,631)

Mostly Rural, Mid-SES	7,837	322 (310,334)	333 (319,346)
Rural, Mid/Low SES	499	377 (337,418)	389 (345,433)
Young, Urban, Mid/High SES	1,022	254 (231,277)	253 (229,278)
Rural, American Indian	437	432 (381,484)	556 (428,684)
Rural, High SES	4,448	457 (438,476)	471 (448,493)
P-value		<0.001	<0.001
Correctional facility CWSs	192	294 (234,354)	277 (219,334)
P-value		0.058	0.043

P-values are from non-parametric Kruskal-Wallis test. [a]CWSs served by groundwater include those served by surface water under the influence of groundwater and groundwater under the influence of surface water. [b]Categories of population served are standard U.S. EPA categories. Population served is adjusted total population served, which accounts for systems that sell or purchase water and avoids overcounting. [c]Very few CWSs served more than one county; of these, approximately half served counties categorized to different sociodemographic county-clusters (e.g., NY7003493 serves New York, New York (Young, Urban, Mid/High SES) and Bronx, New York (Semi-Urban, Hispanic). Sociodemographic clusters were classified based on Wallace et al. (2019).[21] These CWSs are represented for each county that they serve in the sociodemographic county-cluster analyses (N = 32,653). States included in geologic regions are: Alaska/Hawaii (AK, HI), Central Midwest (ND, SD, NE, KS, MO), Eastern Midwest (WI, IL, IN, MI, OH, MN, IA), Mid-Atlantic (PA, MD, DC, DE, NY, NJ, CT, RI), New England (MA, VT, NH, ME), Pacific Northwest (WA, OR, MT, WY, and ID), Southeast (OK, AR, LA, MS, AL, FL, GA, TN, KY, SC, NC, VA, WV), and Southwest (CA, NV, UT, CO, AZ, NM, TX).

Supplemental Table 2. County-level mean estimated community water system (CWS) fluoride concentrations and sociodemographic characteristics for all counties excluded from the analysis (counties with < 100 residents of each racial/ethnic group).

Counties excluded from analysis because the number of residents was <100 for each of the following racial/ethnic groups:

	**non-Hispanic Black**	**American Indian/ Alaskan Native**	**Hispanic/Latino**	**non-Hispanic White**
N	619	190	837	2
**CWS fluoride concentration, µg/L (mean, SD)**	600 (584)	578 (625)	606 (601)	600 (584)
**Sociodemographic characteristics**				
Population size, thousands (mean, SD)	10271 (8218)	5258 (3943)	14488 (12442)	266 (243)
Population density (mean, SD)[a]	16 (18)	11 (14)	39 (127)	0 (0)
% public drinking water sourced from groundwater supplies (mean, SD)[b]	80 (35)	83 (35)	78 (37)	100 (0)
CDC/ATSDR socioeconomic vulnerability index score (mean, SD)	0.39 (0.27)	0.37 (0.29)	0.48 (0.29)	0.7 (0.11)
Median household income (mean, SD)	42632 (7944)	40569 (7331)	41398 (8302)	40528 (8028)
% adults with high school diploma (mean, SD)	87 (10)	86 (12)	86 (9)	*NA (missing)*
% population living in rural area (mean, SD)	82 (24)	97 (10)	79 (24)	100 (0)
**Racial/ethnic composition (mean, SE)**				
% non-Hispanic Black	0 (0)	4 (15)	7 (15)	1 (1)
% American Indian/Alaskan Native	3 (10)	2 (9)	0 (0)	3 (2)
% Hispanic/Latino	8 (16)	2 (2)	8 (16)	49 (39)
% non-Hispanic White	87 (18)	90 (17)	83 (20)	47 (37)

[a] Population density is calculated as number of residents per square mile. [b] The percent of public drinking water sourced from groundwater supplies was calculated using nationwide estimates of water use published by the US Geological Survey for 2010.

Supplemental Table 3. Results from sensitivity analyses evaluating the geometric mean ratio of county-level CWS fluoride concentrations per higher proportions of residents in a given racial/ethnic subgroup, assessing A) 95th percentile county-level fluoride concentrations, B) per 60% higher proportion of residents in a given racial/ethnic group, and C) with adjustment for median household income and the percent of adults with a high school education. Spatial autocorrelation was modeled in spatial error models with autoregressive correlation structure. Model 1 adjusts for population density, the percent of public water sourced from groundwater supplies, and socioeconomic vulnerability score index. Model 2 further adjusts for the percent of public water that was fluoridated.

	A. GMR of 95th percentile county-level fluoride concentrations per 10% higher proportion of residents		B. Per 60% higher proportion of residents of the specific racial/ethnic subgroup	C. Adjusting for median household income and the percent of adults with a high school education, rather than socioeconomic vulnerability score
	**N**	**GMR (95% CI)**	**GMR (95% CI)**	**GMR (95% CI)**
Hispanic/Latino				
Model 1	1918	1.23 (1.13, 1.34)	3.95 (2.43, 6.41)	1.21 (1.12, 1.31)
Model 2		1.18 (1.08, 1.29)	2.80 (1.77, 4.42)	
non-Hispanic Black				
Model 1	1489	1.01 (0.90, 1.13)	1.12 (0.60, 2.09)	1.01 (0.91, 1.11)
Model 2		0.98 (0.87, 1.10)	0.89 (0.49, 1.65)	
American Indian/Alaskan Native				
Model 1	1271	1.08 (0.92, 1.26)	1.26 (0.59, 2.69)	1.03 (0.91, 1.17)
Model 2		1.01 (0.80, 1.27)	1.73 (0.68, 4.42)	
non-Hispanic White				
Model 1	2106	0.83 (0.77, 0.89)	0.34 (0.23, 0.52)	0.88 (0.83, 0.94)
Model 2		0.87 (0.81, 0.94)	0.46 (0.31, 0.67)	

Supplemental Table 4. **Geometric mean ratios (GMR) and 95% CI of county-level community water system (CWS) fluoride concentrations per a 10% higher proportion of residents who are non-Hispanic Black, American Indian/Alaskan Native, Hispanic/Latino, or non-Hispanic White, stratified by region.** Spatial autocorrelation was modeled in spatial error models with autoregressive correlation structure. Models adjusts for population density, the percent of public water sourced from groundwater supplies, socioeconomic vulnerability score index, and the percent of public water that was fluoridated. We did not evaluate models restricted to Alaska/Hawaii because of very small sample sizes. Findings should be considered exploratory as sample sizes were small and effect estimates are unstable, especially for analyses with less than 100 counties and all analyses for the percent of residents who are American Indian/Alaskan Native (county-level percentages of American Indian/Alaskan Native residents were small nationwide).

% Hispanic/Latino	N	GMR (95% CI)	% non-Hispanic Black	N	GMR (95% CI)
Central Midwest	233	1.23 (1.04, 1.45)	Central Midwest	113	1.08 (0.77, 1.52)
Eastern Midwest	359	1.14 (0.79, 1.66)	Eastern Midwest	253	0.57 (0.34, 0.95)
Mid-Atlantic	134	4.30 (2.30, 8.02)	Mid-Atlantic	132	3.39 (1.73, 6.66)
New England	48	0.61 (0.10, 3.54)	New England	46	1.50 (0.04, 58.62)
Pacific Northwest	161	1.47 (0.72, 3.00)	Pacific Northwest	76	156.44 (0.07, 362761.17)
Southeast	601	0.99 (0.64, 1.55)	Southeast	578	1.10 (0.93, 1.29)
Southwest	382	1.04 (0.98, 1.10)	Southwest	291	0.93 (0.78, 1.11)

% American Indian/Alaskan Native	N	GMR (95% CI)	% non-Hispanic White	N	GMR (95% CI)
Central Midwest	122	1.09 (0.97, 1.23)	Central Midwest	320	0.81 (0.72, 0.92)
Eastern Midwest	215	1.08 (0.84, 1.39)	Eastern Midwest	393	1.05 (0.85, 1.29)
Mid-Atlantic	107	0.04 (0.00, Inf)	Mid-Atlantic	135	0.50 (0.37, 0.69)
New England	42	5.24 (0.20, 134.31)	New England	50	0.83 (0.18, 3.75)
Pacific Northwest	134	0.25 (0.06, 1.00)	Pacific Northwest	188	0.75 (0.39, 1.45)
Southeast	390	0.46 (0.03, 8.53)	Southeast	633	0.86 (0.73, 1.02)
Southwest	261	0.42 (0.26, 0.68)	Southwest	387	1.02 (0.96, 1.09)
